# Microstrip Antenna with High Gain and Strong Directivity Loaded with Cascaded Hexagonal Ring-Shaped Metamaterial

**DOI:** 10.3390/ma14237289

**Published:** 2021-11-28

**Authors:** Cheng Cui, Yingnan Ren, Pengfei Tao, Binzhao Cao

**Affiliations:** 1 Department of Physics and Optoelectrons, Taiyuan University of Technology, Taiyuan 030024, China; cuicheng0848@link.tyut.edu.cn (C.C.); renyingnan0756@link.tyut.edu.cn (Y.R.); taopengfei1179@link.tyut.edu.cn (P.T.); 2 Key Laboratory of Advanced Transducers and Intelligent Control System, Ministry of Education, Taiyuan University of Technology, Taiyuan 030024, China

**Keywords:** electromagnetic metamaterial, microstrip antenna, high gain, high directivity

## Abstract

A new cascaded hexagonal ring-shaped metamaterial element is designed, which is arranged periodically and placed on the top of a traditional microstrip antenna to optimize the performance of the traditional antenna. The simulation results show that the new metamaterial microstrip antenna works at near 10 GHz, the impedance bandwidth is extended by 0.25 GHz and the gain is increased by 113.6% compared with a traditional microstrip antenna. Cross-shaped slots are etched on the ground plate of the microstrip antenna to widen the impedance bandwidth. It is shown that the impedance bandwidths at the resonant frequencies of 10 GHz and 14 GHz are broadened by 0.06 GHz and 0.56 GHz, respectively, and the gain of the slot-etched antenna is 13.454 dB. After the metamaterial unit structure is optimized, a nested double-hexagon ring-shaped electromagnetic metamaterial unit structure is proposed. The metamaterial slot microstrip antenna operates in two frequency bands of 10 GHz and 14 GHz; the relative bandwidths are increased to 16.9% and 19.4% with two working bandwidths of 1.74 GHz and 4.98 GHz, respectively; and the gain and directivity are also improved compared with the traditional microstrip antenna. The metamaterial unit structure proposed in this paper is of certain reference value for the variety of metamaterial and the application of metamaterial in traditional microstrip antennas.

## 1. Introduction

The microstrip antenna, first proposed by D. A. Deschamps proposed in 1953, is a kind of flat antenna with a wide range of applications. Its structure includes a radiation patch, an antenna substrate, and a ground plate from top to bottom. The radiation patch and the ground plate are both metal cladding, and the antenna substrate is dielectric material. Microstrip antennas are widely used in wireless communications and radar systems thanks to their advantages of low profile, light weight, low cost, and mass production. However, the traditional microstrip antenna has some shortcomings, such as narrow impedance bandwidth, low gain, and poor directivity, which makes it difficult to satisfy the demand of modern communication equipment. As metamaterials (MTM) exhibit excellent physical properties, microstrip antennas based on metamaterials have become one of the research hotspots [1,2,3].

The electromagnetic properties of metamaterials, artificially arranged and synthesized sub-wavelength structures, can be modulated by adjusting the element size and structure model so as to achieve effective control of electromagnetic waves [4]. In 2000, D. R. Smith et al. successfully developed left-handed metamaterials for the first time, based on previous ideas and methods. Since then, the research on left-hand materials has attracted more scholars’ attention and a variety of left-handed material units with different structures have been proposed, such as Ω type [5], S type [6], I type [7], metal structure [8], triangle [9], cross-ring type [10], and II type [11]. Left-handed metamaterials have many special properties that general materials do not have [12,13,14] and therefore they are often used to make “stealth cloaks” [15,16], improve antenna performance [17,18], and make perfect lenses [19,20] and absorbers [21,22]. Studies have shown that the application of left-handed metamaterials in antenna design can improve the gain of antennas, reduce the coupling between antennas, and miniaturize antennas without compromising other performance [23,24]. In addition, electromagnetic metamaterials do not increase the antenna aperture too much in the antenna design, which will meet the development requirements of high performance, integration, and light weight of radiation devices in the future.

At present, scholars mainly study the metamaterial microstrip antennas from three aspects: high gain [25], high directivity [26], and ultra-wideband [27]. In 2016, Bo Ma et al. designed a double-cross-shaped electromagnetic metamaterial structure unit and placed it as an overlying layer on a traditional microstrip antenna [28]. Compared with the traditional microstrip antenna, the gain at the operating frequency of the metamaterial microstrip antenna was increased from 7.46 dB to 12.35 dB. In the same year, Xiang-Jun Gao et al. proposed a double-layer symmetric single-ring resonator with a negative permeability metamaterial unit [29]. The performance of antennas was improved by arranging metamaterial units of different sizes around a traditional microstrip patch antenna in an orderly manner. The simulation results indicated that, compared with the original microstrip antenna, the gain of the metamaterial microstrip antenna increased by at least 2 dB in two operating bands and the half-power beam width (HPBW) decreased by about 20°, which shows excellent radiation performance. In 2018, Liu Min et al. designed a flower-shaped metamaterial structure, in which a flower pattern is etched on the rectangular radiation patch of the microstrip antenna and cross-shaped slots are etched on the ground plate [30]. This metamaterial microstrip antenna radiated directionally on the *x*-axis and worked over a wide frequency range. The simulation results showed that the impedance bandwidth of the metamaterial microstrip antenna, covering the entire frequency band from 3.5 GHz to 11.6 GHz, was 23.8 times larger than that of the traditional microstrip antenna, and the gain was no less than 5 dB. However, although the design of these metamaterial antennas eliminated the shortcomings of traditional antennas, there were still some problems, such as complex structure, thick substrate, and narrow antenna bandwidth.

On this basis, two kinds of metamaterial unit structures have been designed, which are introduced into the traditional microstrip antenna and their performance indexes are studied. Compared with other studies, the left-handed unit proposed in this paper is simple in structure and has obvious gain enhancement.

## 2. Design and Simulation of Antenna

### 2.1. Design of Cascaded Hexagonal Ring-Shaped Structure

**Figure 1:**
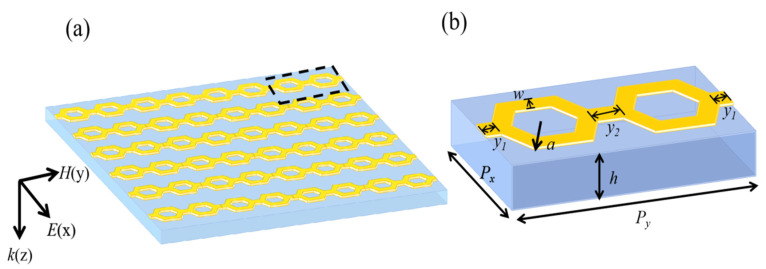
Schematic diagram of cascade hexagonal ring-shaped structure (**a**) Periodic arrangement chart; (**b**) Unit diagram.

The electromagnetic metamaterial unit structure proposed in this section is shown in Figure 1. In the center is a rectangular dielectric substrate, and metal resonators are etched on both sides, which are composed of two hexagonal metal rings with a same radius as a circumscribed circle. The resonance of the metamaterial, at 10 GHz, is produced by adjusting the size and width of the hexagonal metal ring. The metal film and the resonance unit are made of copper and the dielectric layer is made of FR-4 with the dielectric constant *ε*_r_ = 4.4 and the loss tangent tanδ = 0.02. The parameters of the optimized structure are as follows: the dimension of the metamaterial structure unit is *P_x_* = 7.4 mm and *P_y_* = 16.8 mm; the thickness of copper is 0.017 mm; the thickness of the dielectric layer is *h* = 1.6 mm; the radius of the circumscribed circle of the hexagonal metal ring is *a* = 3.7 mm; the width of the hexagonal metal ring is *w* = 2.2 mm; the lengths of the square metal plates are *y*_1_ = 0.5 mm and *y*_2_ = 1 mm, respectively; and the width of the square metal plate is 1 mm.

**Figure 2:**
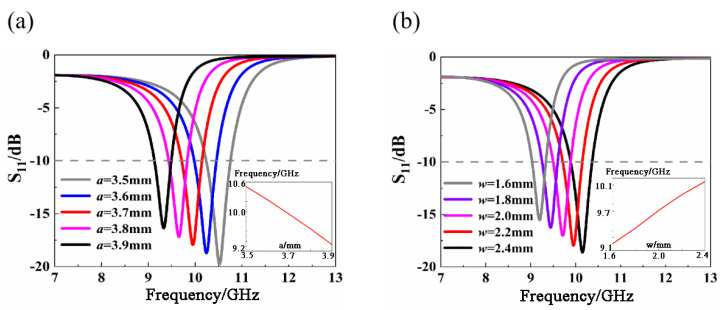
S-parameter curves corresponding to different structural parameters (**a**) When *a* is a different value (**b**) When *w* is a different value.

Figure 2 shows the S-parameter curves for different circumscribed circles of radius a and hexagonal ring widths *w*. It can be seen that with the increase of *a*, the frequency shifts gradually to the left, the depth of resonance decreases, and the bandwidth decreases. With the increase of *w*, the frequency shifts to the right, the resonance depth increases, and the bandwidth increases. So, we chose *a* = 3.7 mm, *w* = 2.2 mm to ensure that the metamaterial structure can be well resonated at 10 GHz while maintaining a wide bandwidth.

In the simulation of the electromagnetic properties of metamaterials, the S-parameter inversion method proposed by D. R. Smith was used to study the properties of metamaterials.

(1) $$n=\frac{1}{kd}cos^{-1}\left(\frac{1-S_{11}^{2}+S_{21}^{2}}{2S_{11}}\right)$$

(2) $$Z=\sqrt{\frac{{\left(1+S_{11}\right)}^{2}-S_{21}^{2}}{{\left(1-S_{11}\right)}^{2}-S_{21}^{2}}}$$

In the formula below, *n* is the refractive index of the dielectric substrate; *d* is the thickness of the dielectric substrate; *S*_11_ is the reflection coefficient; *S*_12_ is the transmission coefficient; and *Z* is the equivalent impedance. According to the reverse solution method, the effective permittivity *ε*_eff_ and equivalent permeability *μ*_eff_ will be deduced.

(3) $$\varepsilon_{eff}=\sqrt{\frac{n}{z}}$$

(4) $$\mu_{eff}=\sqrt{nz}$$

Based on the S-parameter inversion method, the structure was modeled in the simulation software. The electromagnetic wave was perpendicular to the surface of the structure along the *z*-axis, and the ideal magnetic boundary conditions Perfect *E* and Perfect *H* were set along the *x*-axis and *y*-axis, respectively. In this condition, the metamaterial structure can be extended to infinity in the horizontal direction, and the incident, reflected, and transmitted waves are all plane waves. S-parameters were used to predict and verify the existence of the left-handed characteristic frequency band, which is simulated in the frequency range of 7 to 13 GHz.

### 2.2. Cascaded Hexagonal Ring-Shaped Metamaterial Slot Microstrip Antenna

**Figure 3:**
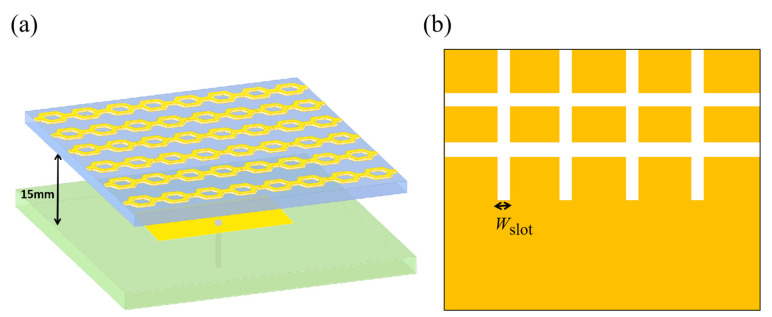
Schematic diagram of a cascaded hexagonal ring-shaped metamaterial slot microstrip antenna (**a**) Three-dimensional view; (**b**) Schematic diagram of the ground plate with etched slots.

Based on the structure unit proposed in the previous section, a cascaded hexagonal ring-shaped metamaterial slot microstrip antenna was designed. Firstly, the reference antenna was designed, and the rectangular patch was selected for the radiation patch. The patch size is parametrically optimized to 8.6 mm in length and 13.2 mm in width to ensure that the center frequency of the microstrip antenna is around 10 GHz. The patch is printed on a PTFE F4B dielectric substrate with a relative dielectric constant of 2.65, a loss tangent of 0.003, and a height of 1 mm. The 50Ω coaxial probe is fed at 2.68 mm on the *x*-axis, away from the center of the patch, to achieve a good impedance match. Both the radiation patch and the ground plate are made of copper with a thickness of 0.017 mm. Secondly, the metamaterial is used as the coating layer, in which the radius of the circumscribed circle of the hexagon is *a* = 4.1 mm; the width of the hexagonal ring is *w* = 2.2 mm; and the hexagonal ring is placed above the reference antenna in the arrangement of 6 × 4 × 1. The size is 70 mm × 70 mm, i.e., 2.33 *λ*_10_ × 2.33 *λ*_10,_ where *λ*_10_ is the wavelength of free space at 10 GHz. The distance between the antenna and the traditional microstrip antenna was 15 mm, i.e., 1/4 *λ*_10_. Cross-shaped slots are etched on the ground plate, and the widths of the slots are *W*_slot_ = 1.3 mm to expand the bandwidth of the antenna. The dual-band metamaterial slot microstrip antenna was designed as shown in Figure 3, where (a) is a three-dimensional view of the metamaterial microstrip antenna, and (b) is a schematic diagram of the ground plate with etched slots.

### 2.3. Nested Double-Hexagon Ring-Shaped Metamaterial Slot Microstrip Antenna

**Figure 4:**
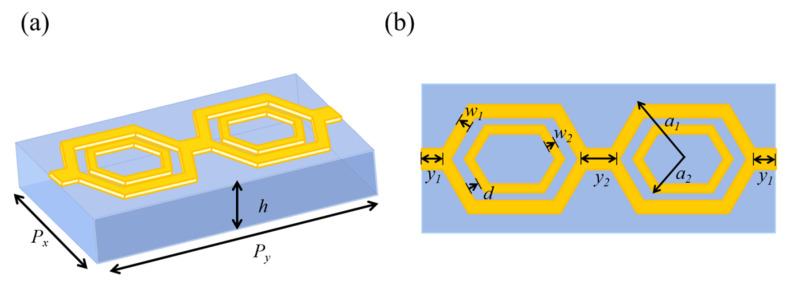
Schematic diagram of nested double-hexagon ring-shaped structure unit (**a**) Structural stereogram; (**b**) Top view of the structure.

A nested double-hexagon ring-shaped electromagnetic metamaterial unit structure is proposed, as shown in Figure 4, to improve the impedance bandwidth of the antenna. In the center is a rectangular dielectric substrate and metal resonators are etched on both sides, which are composed of two large hexagonal rings with the same radius as a circumscribed circle and two small hexagonal rings with the same radius as a circumscribed circle. The two large hexagonal rings are connected by metal plates in the middle and on both sides. The optimized structural parameters are as follows: the dimension of the metamaterial structure unit is *P_x_* = 6 mm and *P_y_* = 14 mm; the thickness of copper is 0.017 mm; the thickness of dielectric layer is *h* = 1.6 mm; the radius of the circumscribed circle of the large hexagonal metal rings is *a*_1_ = 3 mm; the radius of the circumscribed circle of the small hexagonal metal rings is *a*_2_ = 2 mm; the widths of the large hexagonal metal rings are *w*_1_ = 0.3 mm; the widths of the small hexagonal metal rings are *w*_2_ = 0.5 mm; the distance between the large hexagonal metal rings and the small hexagonal metal rings is *d* = 0.7 mm; the lengths of the square metal plates are *y*_1_ = 0.5 mm and *y*_2_ = 1 mm respectively; and the width of the square metal plate is 1 mm.

**Figure 5:**
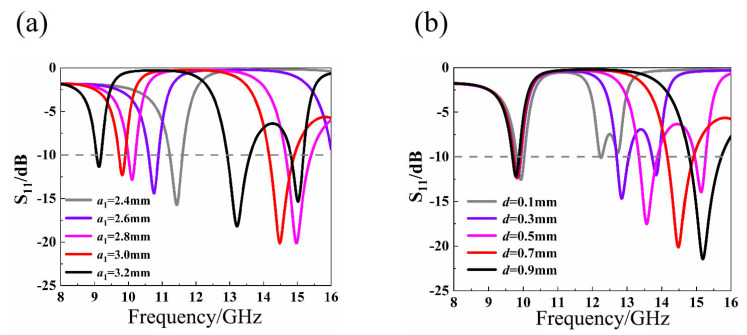

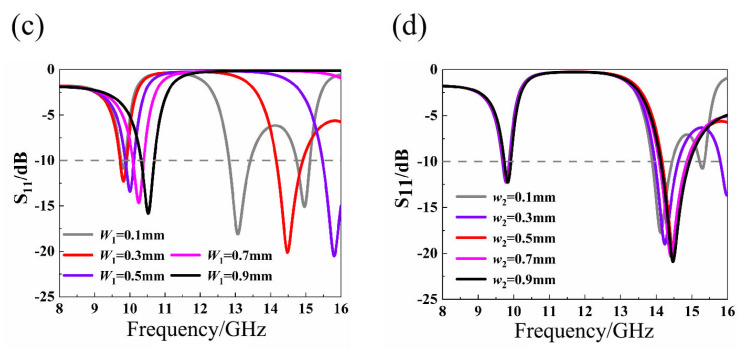
S-parameter curves corresponding to different structural parameter values (**a**) When *a*_1_ is a different value (**b**) When *d* is a different value (**c**) When *w_1_* is a different value (**d**) When *w*_2_ is a different value.

The S-parameter will change with the transformation of metamaterial structure parameter. Figure 5 shows the S-parameter curves of the radius *a*_1_ of the large hexagon ring, the distance *d* between the rings, the width *w_1_* of the large hexagonal ring, and the width *w*_2_ of the small hexagonal ring. Figure 5a,c show that changing *a*_1_ and *w*_1_ will cause the two resonant frequencies to change simultaneously. Figure 5b,d show that changing *d* and *w_2_* only changes the second resonant frequency. Therefore, we chose *a*_1_ = 3.0 mm, *d* = 0.7 mm, *w*_1_ = 0.3 mm, *w*_2_ = 0.5 mm to make the nested double-hexagon toroidal metamaterial structural unit have good resonance at 10 GHz and 14 GHz.

**Figure 6:**
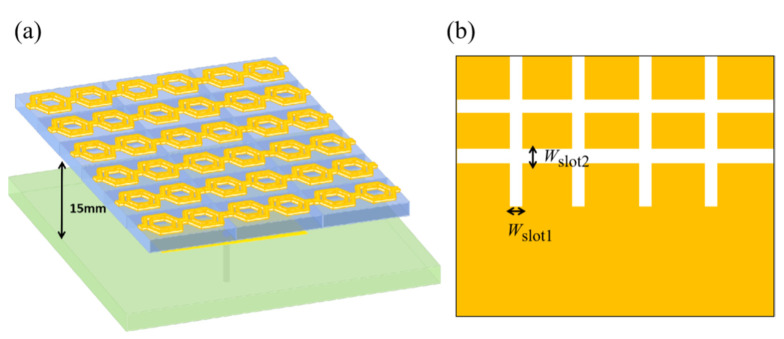
Nested double-hexagon ring-shaped metamaterial slot microstrip antenna (**a**) Three-dimensional view; (**b**) Schematic diagram of etched slots of the ground plate.

A nested double-hexagon ring-shaped metamaterial slot microstrip antenna has been designed based on the unit structure. The structure of the antenna is shown in Figure 6a. The two structure units are connected, with no gap in the transverse direction, and separated by a distance in the longitudinal direction. The radii of the circumscribed circles of the large hexagonal metal rings are *a*_1_ = 3 mm; the radii of the circumscribed circles of the small hexagonal metal rings are *a*_2_ = 2 mm; the widths of the large hexagonal metal rings are *w*_1_ = 0.3 mm; the widths of the small hexagonal metal rings are *w*_2_ = 0.3 mm; and the distance between the large hexagonal metal rings and the small hexagonal metal rings is *d* = 0.7 mm. These rings are placed in a 6 × 3 × 1 arrangement over a traditional antenna as the overlying layer, whose size is 60 mm × 60 mm, i.e., 2 *λ*_10_ × 2 *λ*_10_, where *λ*_10_ is the wavelength of free space at 10 GHz. The vertical distance between the antenna and the conventional microstrip antenna is 15 mm, i.e., 1/4 *λ*_10_. Cross-shaped slots are etched on the ground plate, as shown in Figure 6b, where *W*_slot1_ = 0.5 mm and *W*_slot2_ = 1 mm, to increase the impedance bandwidth of the antenna.

## 3. Simulation Results and Analysis

### 3.1. Property Analysis of Cascaded Hexagonal Ring-Shaped Structure

**Figure 7:**
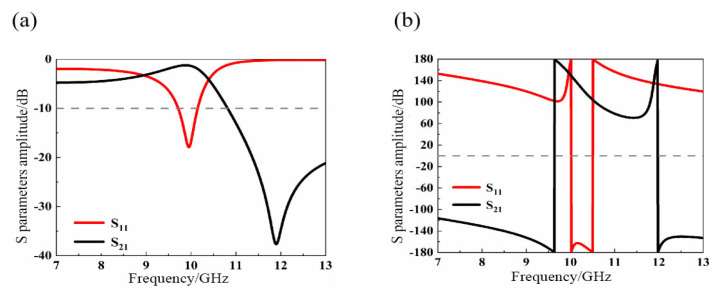
S-parameter curve of cascaded hexagonal ring-shaped structure (**a**) Amplitude graph; (**b**) Phase diagram.

Figure 7 illustrates the reflection coefficient *S*_11_ and transmission coefficient *S*_21_ of the cascaded hexagonal ring-shaped structure unit. The simulation results show that the −10 dB bandwidth is 0.44 GHz; the frequency range is from 9.71 GHz to 11.15 GHz; and the resonant frequency is about 10 GHz. At the resonant frequency, there is a wave trough in *S*_11_ and a wave crest in *S*_21_. It can be seen from Figure 7b that the phases of *S*_11_ and *S*_21_ vary greatly near the resonant frequency, with the phase of *S*_11_ changing from positive to negative and that of *S*_21_ from negative to positive. Therefore, we predict that there might be a left-handed band near 10 GHz.

**Figure 8:**
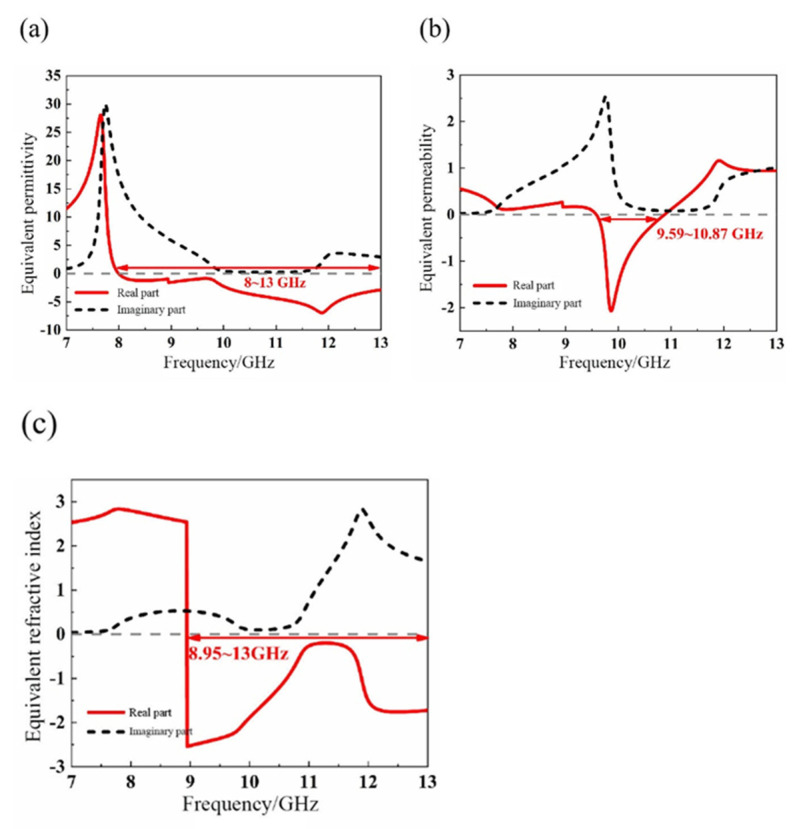
Graph of equivalent electromagnetic parameters (**a**) Equivalent permittivity *ε*; (**b**) Equivalent permeability *μ*; (**c**) Equivalent refractive index *n*.

To confirm this prediction, the method of equivalent parameter extraction is adopted. As shown in Figure 8, the real part of the equivalent permittivity of the structure is negative in the frequency band range of 8~13 GHz, and the real part of the equivalent permeability is negative in the frequency band range of 9.59~10.87 GHz. It can thus be concluded that the left-handed material has double-negative properties in the frequency band range of 9.59~10.87 GHz.

**Figure 9:**
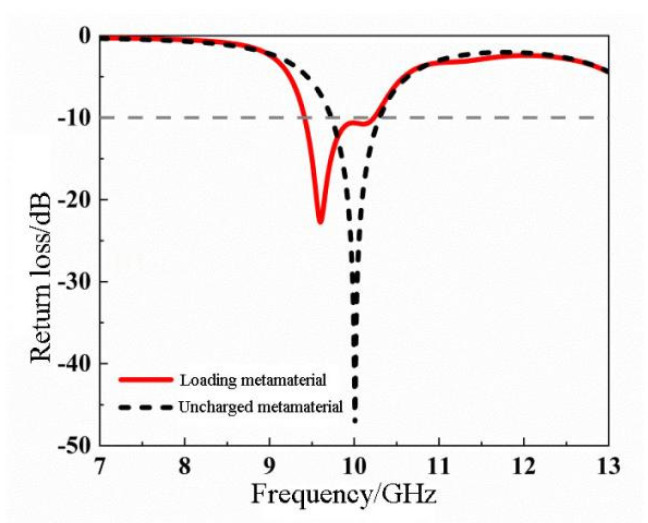
Comparison of the return loss curves of the microstrip antenna loaded with metamaterials and a reference antenna.

As shown in Figure 9, we compared the impedance bandwidth of a cascaded hexagonal ring-shaped metamaterial microstrip antenna with that of a conventional microstrip antenna. After the metamaterial was loaded, the resonant frequency was shifted to the left to 9.6 GHz, and a small new resonance was excited near 10 GHz. Compared with the reference antenna, the impedance bandwidth was increased by 0.25 GHz, which effectively expands the −10 dB impedance bandwidth of the antenna.

**Figure 10:**
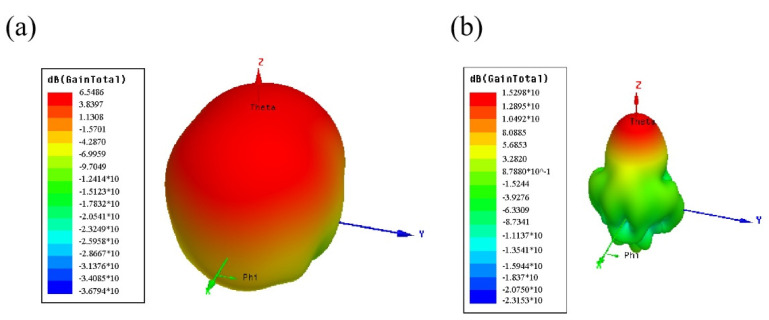
Comparison of the 3D polar coordinate gain of the microstrip antenna loaded with metamaterials and a reference antenna (**a**) Reference antenna; (**b**) Microstrip antenna loaded with metamaterials.

To further explore the performance of the metamaterial microstrip antenna and the traditional antenna, we carried out our research on the gain of the two antennas. As shown in Figure 10a,b, after the metamaterial array was loaded, not only was the impedance bandwidth of the antenna slightly expanded, but also the gain of the antenna was greatly improved. Figure 10a shows that the gain of the reference antenna was 6.5486 dB and Figure 10b shows that the gain of the microstrip antenna was 15.298 dB. The gain at the center frequency of the antenna was increased by 8.7498 dB due to the introduction of the metamaterial array. Due to the fact that the refractive index of the loaded metamaterial element is smaller than that of the dielectric substrate and the radiation energy of the antenna converges to the *z*-axis in the forward radiation direction, the forward radiation gain of the antenna is significantly increased.

**Figure 11:**
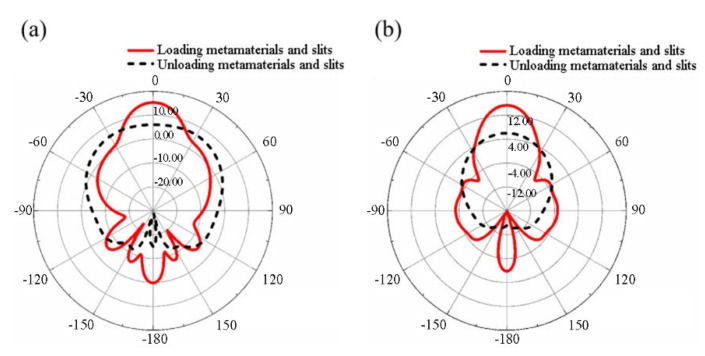
Radiation pattern of a microstrip antenna and a reference antenna loaded with metamaterials (**a**) ***E***-plane (**b**) ***H***-plane.

Figure 11 shows the directional changes of the metamaterial microstrip antenna and the traditional antenna and compares the ***E***-plane and ***H***-plane radiation patterns of the two antennas at the 10 GHz frequency point. It was found that, after the introduction of metamaterials, the directivity of the main beam was significantly enhanced although the side lobe and rear lobe radiation of the antenna was increased. In the *z*-axis direction, both the ***E***-planes and ***H***-planes achieved the maximum radiation, and the pattern shows good symmetry. In addition, the beam width was compressed and narrowed, showing good directional radiation performance, which helps to radiate a longer distance in the single direction of the *z*-axis. The half-power beam width of the ***E***-plane was decreased from 114°to 24°, while the half-power beam width of the ***H***-plane was decreased from 60°to 24°. It can be concluded that, after the introduction of metamaterials, the radiation of the antenna in the zero-degree forward radiation direction becomes concentrated and the antenna has better directional radiation performance.

### 3.2. Two Types of Cascaded Hexagonal Ring

In order to enhance the impedance bandwidth of the antenna, the original cascaded hexagonal ring-shaped structure was improved, and a nested double-hexagon ring-shaped electromagnetic metamaterial unit structure is proposed.

When simulating the designed nested double-hexagonal ring metamaterial structure, the setting was the same as that of the cascaded hexagonal ring metamaterial unit. The electromagnetic wave was perpendicular to the surface of the structure along the *z*-axis, and the ideal magnetic boundary conditions Perfect E and Perfect H were set along the *x*-axis and *y*-axis, respectively. The scattering parameters were simulated in the frequency sweep range of 8~16 GHz, and the S-parameters obtained by the simulation were used to predict and verify the existence of the left-hand frequency band.

**Figure 12:**
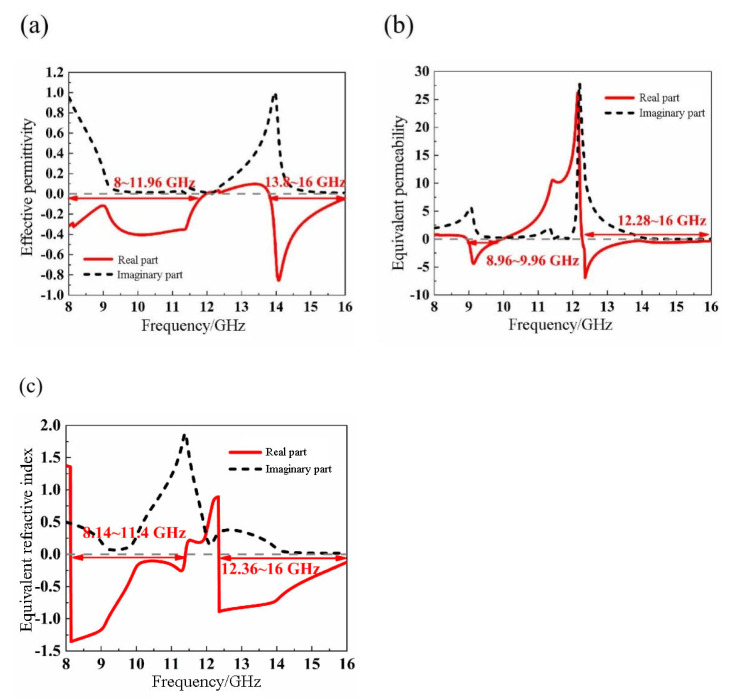
Graph of equivalent electromagnetic parameters extraction (**a**) Equivalent permittivity ε (**b**) Equivalent permeability μ. (**c**) Equivalent refractive index *n*.

Figure 12 shows the equivalent electromagnetic parameters. The results indicate that the real part of the equivalent permittivity of the structure is negative in the two frequency bands of 8~11.96 GHz and 13.8~16 GHz. The real part of the equivalent permeability is negative in the two frequency bands of 8.96~10.96 GHz and 12.28~16 GHz. Therefore, the real parts of the equivalent permittivity and permeability are both negative, in the ranges of 8.96~10.96 GHz and 13.8~16 GHz. Meanwhile, the real part of the equivalent refractive index *n* is negative in the frequency bands of 8.14~11.4 GHz and 12.36~16 GHz, where the peak frequency is 8.12 GHz, and the peak value is 1.35. As a result, the nested double-hexagon ring is a left-handed material with double-negative properties, and the left-handed properties in two frequency bands near at 10 GHz and 14 GHz are verified.

**Figure 13:**
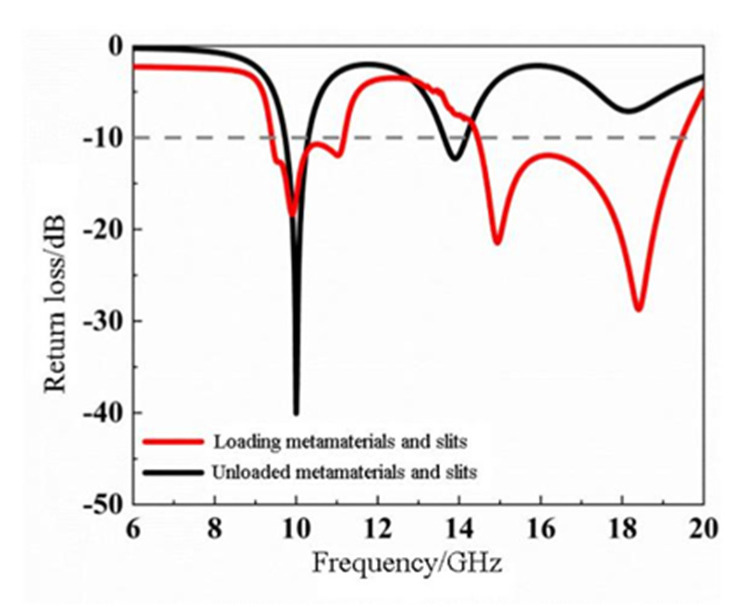
Comparison of the return loss curves of a nested double-hexagon ring-shaped metamaterial slot microstrip antenna and a reference antenna.

To obtain better effect of impedance bandwidths, the research was carried out by loading metamaterials and etching slots. As shown in Figure 13, when the metamaterial antenna operates near at 10 GHz and 14 GHz, the two operating bandwidths are 1.74 GHz and 4.98 GHz and the relative bandwidths of the metamaterial antenna increase to 16.9% and 19.4%, respectively. The results indicate that replacing the metamaterial structure unit and loading slots can effectively expand the operating band of the antenna.

**Figure 14:**
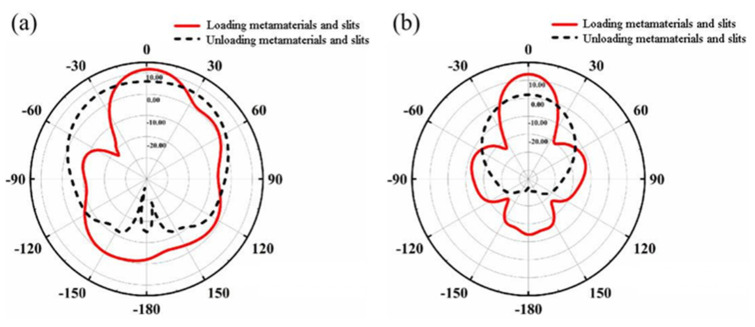
Radiation patterns of a nested double-hexagon ring-shaped metamaterial slot antenna and a reference antenna (**a**) ***E***-plane (**b**) ***H***-plane.

The directivity change of the metamaterial microstrip antenna loaded with metamaterials and slots was compared to that of reference antenna, as shown in Figure 14. For example, in analyzing the far-field radiation patterns of ***E***-plane and ***H***-plane of the two antennas, it can be seen that after the introduction of metamaterials and slots, at 10 GHz, the beam width of the main beam obviously narrows, although the side lobe of the ***E***-plane pattern increases. In addition, the width of the ***H***-plane main beam narrows significantly compared with that of the reference antenna. The main beam is located on the *z*-axis, showing better directional radiation performance. It was found that the radiation of the antenna becomes concentrated in the zero-degree forward radiation direction, which can make the antenna radiate along the *z*-axis.

Therefore, it can be concluded that the nested double-hexagon ring-shaped structure presents more left-handed properties than the cascaded hexagon ring-shaped structure in a multi-frequency band, and such a structure enhances the impedance bandwidth of the antenna and has good directional radiation performance.

## 4. Conclusions

In this paper, a new metamaterial unit that overcomes the shortcomings of traditional microstrip antennas, such as low gain, poor directivity, and narrow bandwidth, has been designed. The simulation results show that the gain is up to 113.6%, which indicates that the structure has excellent performance. Meanwhile, the double-hexagon ring-shaped metamaterial slot microstrip antenna is proposed as an innovation that can increase the relative bandwidth to 16.9% and 19.4% at 10 GHz and 14 GHz, respectively. The metamaterial unit structure proposed in this paper provides new ideas and methods for the variety of metamaterials and the application of metamaterials in traditional microstrip antennas.

## Data Availability

The data that support the findings of this study are available from the corresponding author upon reasonable request.
